# Integrated care at home reduces unnecessary hospitalizations of community-dwelling frail older adults: a prospective controlled trial

**DOI:** 10.1186/s12877-017-0449-9

**Published:** 2017-02-14

**Authors:** Laura Di Pollina, Idris Guessous, Véronique Petoud, Christophe Combescure, Bertrand Buchs, Philippe Schaller, Michel Kossovsky, Jean-Michel Gaspoz

**Affiliations:** 1 Division of Primary Care Medicine, Department of Community Medicine, Primary Care and Emergency Medicine, Geneva University Hospitals, 1, avenue Calas, Geneva, 1206 Switzerland; 2 Department of Ambulatory Care and Community Medicine, University of Lausanne, Lausanne, Switzerland; 3 Clinical Research Centre and Division of Clinical-Epidemiology, Department of Health and Community Medicine, University of Geneva and Geneva University Hospitals, Geneva, Switzerland; 4 Institution genevoise de maintien à domicile (IMAD), Carouge, Switzerland; 5 Association des médecins genevois (AMG), Geneva, Switzerland; 6 Cité générations, Onex, Switzerland

**Keywords:** Aged, Community based interventions, Home care, Chronic disease, Palliative care

## Abstract

**Background:**

Care of frail and dependent older adults with multiple chronic conditions is a major challenge for health care systems. The study objective was to test the efficacy of providing integrated care at home to reduce unnecessary hospitalizations, emergency room visits, institutionalization, and mortality in community dwelling frail and dependent older adults.

**Methods:**

A prospective controlled trial was conducted, in real-life clinical practice settings, in a suburban region in Geneva, Switzerland, served by two home visiting nursing service centers. Three hundred and one community-dwelling frail and dependent people over 60 years old were allocated to previously randomized nursing teams into Control (*N* = 179) and Intervention (*N* = 122) groups: Controls received usual care by their primary care physician and home visiting nursing services, the Intervention group received an additional home evaluation by a community geriatrics unit with access to a call service and coordinated follow-up. Recruitment began in July 2009, goals were obtained in July 2012, and outcomes assessed until December 2012. Length of follow-up ranged from 5 to 41 months (mean 16.3). Primary outcome measure was the number of hospitalizations. Secondary outcomes were reasons for hospitalizations, the number and reason of emergency room visits, institutionalization, death, and place of death.

**Results:**

The number of hospitalizations did not differ between groups however, the intervention led to lower cumulative incidence for the first hospitalization after the first year of follow-up (69.8%, CI 59.9 to 79.6 versus 87 · 6%, CI 78 · 2 to 97 · 0; *p* = .01). Secondary outcomes showed that the intervention compared to the control group had less frequent unnecessary hospitalizations (4.1% versus 11.7%, *p* = .03), lower cumulative incidence for the first emergency room visit, 8.3%, CI 2.6 to 13.9 versus 23.2%, CI 13.1 to 33.3; *p* = .01), and death occurred more frequently at home (44.4 versus 14.7%; *p* = .04). No significant differences were found for institutionalization and mortality.

**Conclusions:**

Integrated care that included a home visiting multidisciplinary geriatric team significantly reduced unnecessary hospitalizations, emergency room visits and allowed more patients to die at home. It is an effective tool to improve coordination and access to care for frail and dependent older adults.

**Trial registration:**

Clinical Trials.gov Identifier: NCT02084108. Retrospectively registered on March 10^th^ 2014.

## Background

Major prolongation of life expectancy has led to a rapid increase in the number of older adults with multiple chronic conditions and dependency [1]. Improving the ability of health care systems to provide high-quality cost-effective health care for this population is a major challenge. It will require a paradigm shift from episodic, short-term interventions characteristic of acute care, to long-term, comprehensive care [2]. Furthermore, the needs and disabilities of frail elders are frequently not identified [3–5]. Fragmentation and discontinuity of care within and between health and social sectors results not only in frequent and unnecessary hospitalizations and emergency room visits, but also in premature nursing home placement with increased health care costs [6]. A large proportion of these patients die unnecessarily in hospital [7–10].

There has been an increasing interest to test whether integrated models of care can improve health outcomes and to shift service utilization from institutions toward communities. Enabling older adults to remain at home has become a priority of government policy in many countries [11].

Depending on the level of collaboration between administrative, financial, and service sectors, three models of integration have been described [12, 13] linkage, coordination and full integration, with a continuum in the degree of collaboration ranging from informal to structured, with all health, social, and supportive services included under a single roof.

While some integrated models have improved health status, the results of satisfaction and utilization of resources are inconsistent. This may be explained, in part, by differences in settings, study design, interventions, length of follow-up, financing, and measured outcomes [14–22]. Furthermore, results from trials that evaluated the effect of in-home geriatric assessment and preventive home visitation programs have also been variable [23–36]. Implementation of these programs outside demonstration projects remains a challenge, particularly in a multi-payer system.

We conducted a prospective controlled study performed in real-life clinical practice settings to evaluate the efficacy of formally coordinating existing resources: 2 home visiting nursing service centers (HVNS) and a community geriatric unit (CGU) that included a physician to perform in-home multidimensional geriatric assessment, and a 24h/ 7 days a week call service for frail older adults. We hypothesized that this approach could decrease the number of hospitalizations, decrease or delay unnecessary hospitalizations, emergency room visits, and institutionalization, as well as increase the probability of respecting care goals of patients with advanced illness who wish to remain at home.

## Methods

### Study design and setting

This prospective controlled trial was performed in two neighboring communities of the Canton of Geneva: Onex (population 17,942) and Bernex, (population 9,772). The socioeconomic status of this population is homogeneous, and 16% are over 65 years old. Two home visiting nursing service centers (HVNS) serve this region, each staffed with two nursing teams (NT) of similar size and composition (eight nurses per team), who provide home visits to patients. Patients are routinely assigned to a NT depending on their place of residence.

### Participants

Participant recruitment began in July 2009 in the Onex HVNS center. Because of difficulty in obtaining recruitment goals the Bernex HVNS center was invited to participate in June 2011. Recruitment goals were obtained in July 2012, and outcomes assessed until December 2012. Consecutive patients 60 years and older who were identified as frail were eligible if followed by a primary care physician (PCP), who had prescribed HVNS services. On the first home visit, nurses routinely performed an initial assessment using items of the Resident Assessment Instrument-Home Care (RAI-HC) [37, 38].

Inclusion criteria were presence of frailty, as defined by one of four alarms or risk factors (impaired cognition, falls, social isolation, or frailty of the informal caregiver support) detected by the RAI-HC. If one risk factor was present, the research nurse calculated a frailty score using the Contact Assessment tool (derived from the InterRAI set of tools) [23]. The nine item frailty measure includes four activities of daily living (bathing, grooming, dressing, walking), two social environment items (living alone and absence of informal caregiver) and three health status items (cognitive impairment, perceived health as poor, and shortness of breath at rest or while performing daily activities). One point is attributed to each affected item, and scores between 1 and 5 reflect mild to moderate frailty, scores ≥ 6 identify severe frailty [39]. Exclusion criteria were patients who did not meet frailty criteria, could not speak French, or were unable to give consent.

#### Sample size calculation and randomization

A sample size of 300 (150 per group) was planned based on a 5% alpha error (two-sided) and a power of 80% to detect a difference in hospitalization rates of 13% per year. This difference was based on a previous meta-analysis of controlled studies that utilized comprehensive geriatric assessment [25]. The expected rate of hospitalization was 30%.

Because of the organization of healthcare delivery, (home visits by nurses), it was not possible to randomize patients individually. In real life clinical setting, each nursing team is responsible for following patients in a predetermined sector, by patient address. To avoid a bias in the allocation of sectors in the control or intervention arm, nurses were randomly assigned in two teams of similar size and composition (eight nurses per team), one team for each arm of the trial. Participants were sequentially allocated into one of the NT groups. Using a random number generator, each NT was designated by a number (1 to 4) and the first four random numbers generated were allocated to the corresponding NT. It was a priori decided that the highest number in each site would be randomized to the intervention and the other to the control group. Blinding was not possible due to the knowledge of the intervention.

#### Control group

Participants (*N* = 179) continued care with their PCP who prescribed services by the HVNS, which include home visits by nurses and nurses’ aides one to three times a day depending on patients’ needs (e.g., administration of medication, measurement of vital signs and glycaemia, wound care, support in activities of daily living) and /or technical support for in-home hospitalization. No formal case management was provided. As not all PCPs perform home visits, participants who required emergency service after office hours were usually instructed to contact a physician-on-call emergency service or go to an emergency room.

#### Intervention group

Participants (*N* = 122) were followed by their PCP, the intervention NT, and additionally provided with in-home geriatric assessment by the community geriatrics unit (CGU) doctor in the following domains: cognition (Mini-Mental State Exam [40] and clock drawing test), mood (Geriatric Depression Scale [41]) functional status (basic [42] and instrumental activities of daily living, [43] gait (timed up and go [44] and semi tandem stand, [45] nutrition (Mini-Nutritional Assessment (short form), [46] pain (visual analogue scale), medication review and adherence. The results were transmitted in writing to the PCP and NT with recommendations, and, in the event of complex issues, meeting between the CGU and NT were organized. Participants and NT received written instructions to first contact the PCP in case of an emergency; if unavailable, the CGU that provided a 24h/7 days a week medical call service was contacted. The CGU home intervention team included doctors, physical and occupational therapists, psychologists, dieticians and social workers. A day hospital is also part of provided services [47].

#### Measures

Data were collected from patient computerized medical and nursing records. The primary outcome measure was the number of hospitalizations. Secondary outcomes were the reason of hospitalization and the number and reason of emergency room visits, institutionalization, death, and place of death.

Reasons for hospitalizations included falls, fractures, acute medical problems, psychiatric problems, or social reasons (unnecessary). Reasons for emergency room visits included falls, acute medical, or psychiatric problems and social reasons (unnecessary). Reason and number of consultations for an acute problem (phone or home visit) by the CGU, PCP, or other emergency service were also collected.

Unnecessary hospitalizations were identified by chart review by the research nurse and defined as those occurring in the absence of an acute medical problem and that could have been handled by a general practitioner or home care program.

#### Statistical analysis

Descriptive results are reported by counts and percentages or by means and standard deviations (sd). Characteristics were compared using the Student test, Chi-square or Fisher exact tests. Primary and secondary outcomes were compared in intention-to-treat by using survival analyses. For mortality, the Kaplan-Meier survival estimator, log-rank test and Cox regression model were used. As death was a competing event for the other outcomes, competing risk models were used [48, 49]. The cumulative incidences and the hazard ratio were obtained with the package *cmprsk* for R version 3.0.1. All reported hazard ratios were adjusted to NT and tests for comparison of cumulative incidence over three-year’ follow-up were stratified by NT. The proportionality of hazards was graphically inspected (plots of the complementary log-log functions of survival). When hazards were not proportional, an interaction between the intervention’s effect and the time was added in the model: the estimates of hazard ratio (HR) could be different between the first year of follow-up and after the first year. The cumulative incidences were compared at one, two, and three years using a Wald test. Two-sided *p*-values less than 0.05 were considered significant. All analyses were performed using S-Plus 8.0 for Windows (Insightful Corp., Seattle, WA) and R version 3.0.1 (R Development Core Team. *R: A Language and Environment for Statistical Computing.* Vienna, Austria: R Foundation for Statistical Computing; 2010).

## Results

**Fig. 1:**
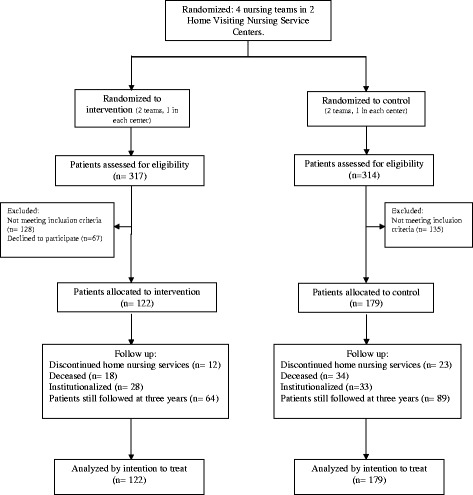
Flow chart

Enrollment, allocation and follow-up are described in Fig. 1. Two hundred and twenty-three participants were recruited in the first two years, 78 in the last 12 months and 4 patients in the last 6 months. 52.5% of subjects in the intervention group and 49.7% in the control group were still in the study at the end of data collection (December 15 2012). Length of follow-up ranged from 5 to 41 months with a mean of 16.3 months and is reported in Table 1. Baseline characteristics are shown in Table 2 and were similar between groups.

**Table 1:** Description of length of follow-up and status at end of study

	Intervention (*n* = 122)		Control (*n* = 179)	
	*N* (%)	Median follow-up in days [IQR]	*N* (%)	Median follow-up in days [IQR]
Death	18 (14.8)	294 (116;555)	34 (19.0)	298 (150;505)
Institutionalization	28 (23.0)	412 (203;818)	33 (18.4)	401 (226;619)
Withdrawal	12 (9.8)	148 (41;340)	23 (12.8)	176 (78;337)
Still in study	64 (52.5)	592 (308;910)	89 (49.7)	435 (291;599)

**Table 2:** Baseline demographic and clinical characteristics

	Intervention (*N* = 122)	Control (*N* = 179)	*P* value
Baseline characteristics			
Female	78/122 (63.9%)	120/179 (67.0%)	0.66
Age			0.99
60–79	49 (40.2%)	70 (39.3%)	
80–89	56 (45.9%)	82 (46.1%)	
90–100	17 (13.9%)	26 (14.6%)	
Mean (sd)	81.8 (8.2)	81.9 (8.2)	0.85
Live alone	32/121 (26.4%)	57/173 (32.9%)	0.29
ADL (0–6), mean (sd)	1.2 (1.6)	1.1 (1.4)	0.52
IADL (0–8), mean (sd)	5.7 (1.8)	5.4 (2.0)	0.23
Perceived health as poor	52/104 (50.0%)	65/161 (40.4%)	0.16
Psychotropic medications	76/112 (67.9%)	93/137 (67.9%)	0.90
Analgesics (opioid + non opioid)	67/115 (58.3%)	79/143 (55.2%)	0.72
BMI <21	25/107 (23.4%)	29/135 (21.5%)	0.85
Alarms (RAI-HC instrument)			
Number of alarms (range 1–4)	2.1 (0.9)	2.1 (0.9)	0.59
Cognition	61/122 (50.0%)	88/179 (49.2%)	0.98
Falls	89/122 (73.0%)	132/179 (73.7%)	0.98
Social isolation	65/122 (53.3%)	87/179 (48.6%)	0.50
Frailty of informal caregiver	46/122 (37.7%)	66/179 (36.9%)	0.98
CA SCORE			
1–5 (mild-moderate frailty)	92/122 (75.4%)	135/179 (75.4%)	0.89
6–9 (severe frailty)	30/122 (24.6%)	44/179 (24.6%)	
Diagnoses			
Cardiac disease	51/113 (45.1%)	54/159 (34.0%)	0.08
Degenerative joint disease	59/113 (52.2%)	97/160 (60.6%)	0.21
Diabetes	28/109 (25.7%)	34/153 (22.2%)	0.61
Obstructive pulmonary disease	27/110 (24.5%)	27/156 (17.3%)	0.20
Cancer	26/112 (23.2%)	39/155 (25.2%)	0.82
Depression	46/108 (42.6%)	55/158 (34.8%)	0.25
Dementia	30/110 (27.3%)	39/156 (25.0%)	0.78
Stroke	19/105 (18.1%)	28/153 (18.3%)	0.90
Chronic renal failure	19/108 (17.6%)	16/152 (10.5%)	0.14
Urinary incontinence	47/121 (38.8%)	57/176 (32.4%)	0.31
Chronic pain	55/87 (63.2%)	75/111 (67.6%)	0.62
Visual impairment	52/110 (47.3%)	63/157 (40.1%)	0.30

Results are reported by counts and percentages or by means and standard deviation (sd)

*Abbreviations*: *ADL* activities of daily living (score reported is number of deficient activities), *IADL* instrumental activities of daily living (score reported is number of deficient activities), *RAI-HC* Resident Assessment Instrument-Home Care, *CA* Contact assessment score

### Hospitalizations

**Fig. 2:**
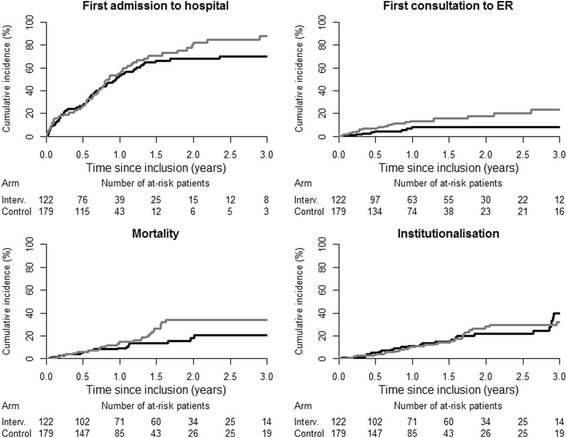
Cumulative incidences for primary and secondary outcomes. First hospitalization rate was not significant different over the three year follow-up period (global *p* = 0.24). However, first hospitalization rate was significantly reduced at two (*p* = 0.04) and three years (*p* = 0.01). First emergency room visit was significantly reduced over the three year follow-up period (global *p* = 0.03). No difference was found in institutionalization rates (global *p* = 0.70). Mortality was not significantly reduced (global *p* = 0.06) over the three year follow-up period. A significant difference appeared at two years (*p* = 0 · 03) with a trend at three years (*p* = 0 · 07) in favor of the intervention arm (see text for details)

Fifty nine percent of participants in the intervention group and sixty percent of participants in the control group had at least one hospitalization (Table 3). The cumulative incidence for a first hospitalization (Fig. 2), was not significantly lower in the intervention than in the control group (*p* = 0.24). However, a difference in favor of the intervention group appeared after the first year (three-year cumulative incidence 69.8% in the intervention group and 87.6% in the control group, *p* = 0.01; see Table 3). In a post-hoc analysis, the HR was 0.94 (95%CI 0.67 to 1.31, *p* = 0.71) over the first year and 0.48 (95%CI 0.24 to 0.95, *p* = 0.04) over the second and third year of follow-up. The HR over the three year follow-up period was 0.82 (95%CI 0.61 to 1.11, *p* = 0.21)

**Table 3:** Main and secondary outcomes

	Intervention (*n* = 122)	Control (*n* = 179)	*P*
Hospitalizations			
No hospitalization^b^	50/122 (41.0%)	73/179 (40.1%)	
One to three hospitalizations^b^	66/122 (54.1%)	95/179 (53.1%)	
Four or more hospitalizations^b^	6/122 (4.9%)	11/179 (6.1%)	
One-year rate of first hosp.^a, b^	53.6 (43.9 to 63.3)	55.4 (47.0 to 63.8)	0.78
Two-year rate of first hosp.^a, b^	67.8 (58.1 to 77.4)	82.3 (72.6 to 92.1)	0.04
Three-year rate of first hosp.^a, b^	69.8 (59.9 to 79.6)	87.6 (78.2 to 97.0)	0.01
Length of stay (days)^c^	37.5 (18.8 to 84.0)	51.0 (26.3 to 93.5)	0.18
Reasons for hospitalizations^d^			0.03
Falls	28/122 (23.0%)	29/205 (14.1%)	0.06
Fracture	6/122 (4.9%)	8/205 (3.9%)	0.20
Acute medical problem	80/122 (65.6%)	132/205 (64.4%)	0.92
Psychiatric problem	3/122 (2.5%)	12/205 (5.9%)	0.18
Unnecessary	5/122 (4.1%)	24/205 (11.7%)	0.03
Emergency room visit (ERV)			
At least one ERV	8/122 (6.6%)	26/179 (14.5%)	0.04
One-year rate of first ERV^a^	8.3 (2.6 to 13.9)	13.4 (7.8 to 19.0)	0.21
Two-year rate of first ERV^a^	8.3 (2.6 to 13.9)	17.8 (10.3 to 25.2)	0.045
Three-year rate of first ERV^a^	8.3 (2.6 to 13.9)	23.2 (13.1 to 33.3)	0.01
Reasons for consultation^e^			0.28
Falls	5/8 (62.5%)	10/34 (29.4%)	0.11
Unnecessary	0/8 (0.0%)	8/34 (23.5%)	0.32
Acute medical problem	3/8 (37.5%)	15/34 (44.1%)	1
Psychiatric	0/8 (0.0%)	1/34 (2.9%)	1
Institutionalization			
One-year rate of placement^a^	11.2 (5.1 to 17.2)	10.3 (5.3 to 15.4)	0.83
Two-year rate of placement^a^	21.6 (12.9 to 30.4)	28.2 (18.4 to 37.9)	0.33
Three-year rate of placement^a^	39.4 (25.1 to 53.6)	31.8 (21.3 to 42.3)	0.40
Mortality			
One-year mortality^a^	9.4 (3.8 to 15.1)	15.0 (8.9 to 21.1)	0.19
Two-year mortality^a^	17.9 (8.9 to 27.0)	33.7 (23.1 to 44.3)	0.03
Three-year mortality^a^	20.4 (10.4 to 30.3)	33.7 (23.1 to 44.3)	0.07
Place of death			
At home	8/18 (44.4%)	5/34 (14.7%)	0.04

^a^cumulative incidences are expressed in percentage and reported with 95% confidence intervals

^b^the rate of first hospitalization rate were assessed using Kaplan-Meier’s approach to account for varying length of follow-up across patients, while the reported percentages of with no, one to three and four or more hospitalizations did not account for length of follow-up and were only descriptive statistics

^c^includes hospitalization in internal medicine and geriatrics and rehabilitation

^d^percentages of the number of hospitalizations, ^e^percentages of the number of visits

Unnecessary hospitalizations for social reasons were significantly less frequent in the intervention group (4.1% versus 11.7%, *p* = 0.03) (Table 3). Length of hospital stay was lower, but not significantly, in the intervention than in the control group (37.5 versus 51.0 days *p* = 0.18).

### Emergency room visits

Eight (6.6%) participants in the intervention and 26 (14.5%) in the control group had at least one emergency room visit, *p* = 0.04 (Table 3). The three-year cumulative incidences were 8.3% in the intervention and 23.2% in the control group, *p* = 0.01 (Fig. 2). The HR over the entire follow-up period for emergency room visits was 0.43 (95% CI 0.19 to 0.94, *p* = 0.04).

### Institutionalization

No difference was found (*p* = 0.70; see Fig. 2 and Table 3), with institutionalization for 28 (23%) in the intervention and 33 (18%) in the control group. HR was 1.05 (95%CI 0.63 to 1.79, *p* = 0.84).

### Mortality

Eighteen (14.8%) participants in the intervention and 34 (19.1%) in the control group died. The cumulative mortality was lower in intervention than in control group but not significantly (*p* = 0.06). A difference in mortality seemed to appear after the first year in favor of the intervention group but was not statistically significant (Fig. 2, Table 3). The HR over the entire follow-up was 0.58 (95%CI 0.32 to 1.05, *p* = 0.07). Death occurred significantly more often at home in the intervention group (44.4 versus 14.7%, *p* = 0.04; Table 3).

### Home consultations for an acute medical problem

Ninety-seven participants in the intervention group (79.5%) presented an acute problem leading to at least one urgent consultation by phone (34.9%) or home visit (65.1%) by the CGU doctor (60.3% of the time) or other emergency services. Ninety-five participants in the control group (53.1%) received at least one urgent consultation, either by telephone (47.7%) or through a home visit (52.3%) by their PCP (58.4% of the times) or another physician-on-call emergency service. On average, participants in the intervention received four times more consultations (telephone or home visit) than the control group (6.3 versus 1.6 per patient, *p* <0.001). Home visits resulted in a hospitalization in 8.7% of the cases in the intervention and in 19% in the control group (*p* = <0.001).

## Discussion

This prospective controlled trial showed that formally coordinating existing resources of the public and private sectors for frail and dependent-community dwelling older adults reduced the rate of hospitalizations after the first year, decreased unnecessary hospitalizations, lowered the rate of emergency room visits after the first year, and increased the proportion of patients dying at home. We did not find differences in the number of total hospitalizations or rates of institutionalization or mortality.

Few studies have included round-the-clock access to a geriatrics team. One such trial [21] included a 22-month follow-up and showed a 50% reduction in hospital alternate level stays with no reduction in emergency room utilization, hospitalization or nursing-home placements.

Although we did not find a global reduction in hospitalizations over the follow-up period, the rate of hospitalizations was decreased already after the first year, suggesting that reduction in these rates may be related to length of follow-up. Unlike other studies that did not record the reason for hospitalization and emergency room visits, we were able to report that these events were mostly justified by an acute illness (most frequently an acute cardiac event, stroke, infections, falls, fractures, psychiatric and behavioral problems in demented patients as well as need for in-hospital palliative care), and, more importantly, that unnecessary hospitalizations due to social reasons, such as lack or breakdown of the informal caretaker system, had decreased. In fact, the intervention arm received four times more consultations at home by the UGC for an acute problem that resulted in a hospitalization in a small number of the cases (8.7%). Although it is possible that this quantitative difference alone may be responsible for some of the results, it is likely that qualitative changes and geriatric expertise were also important in ensuring better primary outcomes and allowing those who wished to die at home. These results suggest that improving collaboration and communication with rapid access to care in case of emergency or after office hours could avoid undesirable outcomes, since this also had a positive effect in the reduction of emergency room visits in the intervention group.

Several trials and meta-analyses have stated that the efficacy of comprehensive geriatric assessment is highly dependent on the number of visits, control over implementation of recommendations, and extended ambulatory follow-up [26–30]. Programs that include integrated models of care have been more likely to be effective [17–23, 35]. Our study is consistent with these findings.

Many reviews of geriatric assessment have suggested to target primarily healthier older populations [33]. We were able to show several positive results of a simple integrated intervention in a particularly old and dependent group. Thus, our findings provide important evidence for the inclusion of highly dependent populations in integrated models of care.

No difference was found between groups in rates of institutionalization. This is consistent with a 2002 meta-analysis of home visit programs that showed reduced nursing-home placement only in high functioning older persons with multiple follow-up visits [30]. Institutionalization is also closely related to bed availability. And, it should be noted that our trial was carried out in a Swiss region with the lowest number of long-term care beds per capita and a long average wait for a nursing-home bed [50].

Although a global effect on mortality was not shown, the significant decrease at two years and the trend at three years suggests that mortality may have been postponed. Reduction of mortality has been reported in an earlier meta-analysis mostly in younger populations [30]. Our results are particularly striking in view of the heavy burden of disease and disability in our sample, and warrants confirmation in larger studies.

Most importantly, the intervention had a marked positive effect on place of death, an issue not reported in most prior trials of in-home geriatric assessment, and reflects the capacity of integrated models to honor individuals’ preferences and deliver quality end-of-life care. Although we considered “dying at home” as a desirable outcome, it should be acknowledged that some people with concrete palliative care needs prefer to be hospitalized before death, We found in our study that most of our patients declared their wish to die at home and were able to carry out this wish in slightly less than half of the intervention cases. This was a significant improvement compared to controls, but suggests that further efforts are needed to increase access to this option. A similar result was reported by the program of All-Inclusive Care for the Elderly (PACE), [9] although not evaluated by a controlled trial; it showed that 45% of participants died at home, compared to 20% in the general geriatric population.

The strengths of our approach include the integration of existing public (HVNS and CGU) and private (PCP) services, the availability of a multidisciplinary geriatrics team, including a round-the-clock medical call service, and a detailed analysis of the reasons for hospitalizations and emergency room visits, as well as place of death. Furthermore, as recommended in a large 2008 meta-analysis, [33] this study was conducted in a real clinical practice setting.

Limitations of our study include that patients were not randomized individually but only to nursing teams. Therefore, we cannot exclude that differences in outcomes between intervention and control arms are potentially caused by a confounding phenomenon. However, patients’ characteristics were similar in both arms, in particular characteristics that captured frailty, hence the risk of a confounding phenomenon related to these characteristics is low. Furthermore, since the intervention and the control nursing teams worked in the same HVNS center, it was not possible to perform a double-blind study and contamination between groups cannot be excluded. The research nurse who collected objective outcomes was independent and not a member of the HVNS center or the CGU and had no stake in the results of the study, further reducing the risk of introducing bias.

The study was initially designed to assess the cumulative incidence of first hospitalization at one year. However, due to lower than expected recruitment, the enrollment period was extended at the first site and a second HVNS center was added with a shorter follow-up than the first. Therefore, we analyzed data up to end of follow-up and also at one year. Although only half the subjects remained in the study upon its termination, most of the drop-outs were due to reached pre-determined end points, such as death or institutionalization, which reflects the severe morbidity burden of the studied population.

We have used a definition of frailty based on the RAI-HC classification system, which has the advantage of being nursing based and is routinely administered on admission. It is, however, slightly different from the more medically-based Fried criteria [51] (for example weight loss and isometric hand strength are not included) and readers should take this into consideration when comparing our results to other studies.

Finally, we encountered several difficulties and temporary interruptions in recruitment during the study period. For example, the Onex center moved their offices to a nearby location at the end of 2010. The Bernex center transitioned to a computerized record system in the summer of 2011. A 5 month maternity leave of the research nurse in 2010 and need to find a replacement was an added reason for recruitment delay. All these events show that promoters of complex interventions in everyday practice should be aware of the possible emergence of unexpected environmental changes.

## Conclusions

This three-year trial showed effectiveness of an integrated care approach that included in-home geriatric assessments performed by a physician, long-term coordinated follow-up, and availability of a round-the-clock geriatric call service, for the care of frail and dependent elderly. This approach led to the following outcomes: a reduction in hospitalization rate after the first year; a reduction in unnecessary hospitalizations due to social problems; a global reduction in emergency room visits; and, an increase in the proportion of patients who could die in their own home. These are important quality-of-life issues for older adults with progressive chronic illness.

We expect these results may be reproducible in other systems since the study was performed in a real life clinical practice setting and utilized existing resources. Finally, these results suggest the need to expand and implement integrated care models, including multidisciplinary geriatrics teams who can provide home visits and call services as a tool to improve long-term, comprehensive care for highly dependent community dwelling older adults.

## Abbreviations

CGU: Community geriatrics unit
HVNS: Home visiting nursing service
NT: Nursing team
PCP: Primary care physician
RAI-HC: Resident assessment Instrument-Home Care
